# The Association of Delayed Care With Depression Among US Middle-Aged and Older Adults During the COVID-19 Pandemic: Cross-sectional Analysis

**DOI:** 10.2196/29953

**Published:** 2021-10-05

**Authors:** Yan Luo

**Affiliations:** 1 The University of Alabama Tuscaloosa, AL United States

**Keywords:** depression, COVID-19, delayed care, middle-aged adults, older adults

## Abstract

**Background:**

During the COVID-19 pandemic, the depression level among US adults has significantly increased. Age disparity in depression during the pandemic has been reported in recent studies. Delay or avoidance of medical care is one of the collateral damages associated with the COVID-19 pandemic, and it can lead to increased morbidity and mortality.

**Objective:**

This study aimed to assess the prevalence of depression and delayed care among US middle-aged adults and older adults during the pandemic, as well as investigate the association of delayed care with depression among those 2 age groups.

**Methods:**

This cross-sectional study used data from the 2020 Health and Retirement Study (HRS) COVID-19 Project (Early, Version 1.0). Univariate analyses, bivariate analyses, and binary logistic regression were applied. US adults older than 46 years were included. Depression was measured by the Composite International Diagnostic Interview-Short Form (CIDI-SF). Delayed care was measured by the following 4 items: delayed surgery, delayed seeing a doctor, delayed dental care, and other delayed care.

**Results:**

A total of 3246 participants were identified. More than half of the participants were older than 65 years (n=1890, 58.2%), and 274 (8.8%) participants had depression during the pandemic. Delayed dental care was positively associated with depression among both middle-aged adults (OR 2.05, 95% CI 1.04-4.03; *P*=.04) and older adults (OR 3.08, 95% CI 1.07-8.87; *P*=.04). Delayed surgery was positively associated with depression among older adults (OR 3.69, 95% CI 1.06-12.90; *P*=.04). Self-reported pain was positively related to depression among both age groups. Middle-aged adults who reported higher education levels (some college or above) or worse self-reported health had a higher likelihood of having depression. While perceived more loneliness was positively associated with depression among older adults, financial difficulty was positively associated with depression among middle-aged adults.

**Conclusions:**

This study found that depression was prevalent among middle-aged and older adults during the pandemic. The study highlighted the collateral damage of the COVID-19 pandemic by identifying the association of delayed surgery and dental care with depression during the pandemic. Although surgery and dental care cannot be delivered by telehealth, telehealth services can still be provided to address patients’ concerns on delayed surgery and dental care. Moreover, the implementation of telemental health services is needed to address mental health symptoms among US middle-aged and older adults during the pandemic. Future research that uses more comprehensive measurements for delayed care is needed to decipher the path through which delayed care is associated with depression.

## Introduction

### COVID-19 Outbreak

Since first recognized in December 2019, COVID-19 has posed significant challenges for public health, research, and medical communities [1,2]. On March 11, 2020, the World Health Organization announced that COVID-19 can be characterized as a pandemic [3]. During the pandemic, most states in the United States have taken nonpharmaceutical pandemic control measures, including imposing mandatory stay-at-home orders; closing or limiting capacity at nonessential businesses, restaurants, and bars; closing schools; limiting large gatherings; requiring quarantines; and requiring masks [4,5].

### Depression During the Pandemic

Comparing national representative data collected before and during the pandemic, prior studies have demonstrated that the depression level among US adults significantly increased during the pandemic [6,7]. Daly et al used Patient Health Questionnaire-2 (PHQ-2) and found that the rate of US adults with depression was 5.7% higher during the pandemic compared to the rate in 2017-2018 [6]. Ettman et al applied Patient Health Questionnaire-9 (PHQ-9) and reported that the prevalence of depressive symptoms among US adults was 3-fold higher during the pandemic compared to before the pandemic [7]. Even without the comparison, recent studies used different depression measurements and reported high levels of depression among US adults during the pandemic [8-11]. The worsened depression level among US adults might be related to the nonpharmaceutical measures taken to control the pandemic. Those measures had significant social and economic consequences that might in return harm health [4]. For example, school closure caused disrupted educational development, social distancing caused lack of access to social support systems, and business closure caused unemployment [4]. In fact, job insecurity, unemployment, loneliness, and social support were cited as factors associated with depression among US adults during the pandemic [7,8,12-14].

### Age Disparity in Depression

Age disparity in depression during the pandemic was reported in recent studies [15-18]. While older age is associated with a higher risk of COVID-19 infection and worse outcomes [19,20], younger age is a risk factor for a higher level of depression during the pandemic [15-18]. Previous studies found that loneliness, COVID-19–specific worries, job insecurity, resilience, and social support were significantly related to depression levels among young US adults aged 18 to 35 years during the pandemic [8,12,13]. Studies examining the factors associated with depression among middle-aged and older adults during the pandemic were not found. However, previous studies indicated that while depression in middle-aged adults might be related to lifecycle gains and losses, marriage, employment, and economic well-being [21], depression in older adults is often linked to coexisting medical conditions or cognitive impairment [22].

### Delayed Care and Depression During the Pandemic

Delay or avoidance of medical care is one of the collateral damages caused by the COVID-19 pandemic, and it can lead to increased morbidity and mortality [23,24]. A nationwide survey conducted among US adults from June 24 to 30, 2020, indicated that approximately 40.9% of US adults reported avoidance of medical care during the pandemic because of COVID-19 concerns [25]. A recent study found a significant association between depression and delayed medical care among US adults amidst the pandemic [26]. Delayed medical care also might lead to untreated or undertreated pain [27,28] that was found to be associated with significantly higher depression levels compared to the time when pain was treated [29].

### Aim

While depression and its associated factors among young adults during the pandemic have been well studied [8,12,13], studies focusing on the prevalence of depression and its linked factors among middle-aged and older adults during the pandemic are scarce. Moreover, as a population that is more likely to have medical conditions [30], older adults were disproportionally impacted by delayed care during the pandemic because their existing medical conditions might have been untreated or undertreated [27,28]. Because depression in older adults is often related to their coexisting medical conditions or cognitive impairment [22], the pandemic might pose a threat to older adults’ mental health status through increased delayed care. Previous studies have not investigated the association of delayed care with depression among US middle-aged and older adults during the pandemic. Using national representative survey data, this study aimed to (1) assess the prevalence of depression and delayed care among US middle-aged adults (46-64 years old) and older adults (≥65 years old) during the COVID-19 pandemic and (2) investigate the association of delayed care with depression among these 2 age groups during the pandemic. In line with the Medicare-eligible age for older adults, 65 years was used as a cutoff to differentiate older adults from middle-aged adults [31].

## Methods

### Data Description

This study used the 2020 Health and Retirement Study (HRS) COVID-19 Project (Early, Version 1.0) data that are part of the HRS, which is sponsored by the National Institute on Aging (grant number NIA U01AG009740) and is conducted by the University of Michigan [32]. The HRS is a national longitudinal study collecting data on economy, health, marital status, and family status, as well as support systems among older Americans [32]. The HRS sampled at the household level and built the sample over time [33]. Since the first wave of the HRS in 1992, a new cohort of individuals aged 51 to 56 years has been added every 6 years (eg, in 1998, 2004, 2010, and 2016) [34]. If the person meeting the age eligibility was coupled, their spouse or partner was also included in the sample [33]. The COVID-19 sample was randomly selected from households who were originally assigned to enhanced face-to face interviewing (EFTF) and then split into the following 2 random samples: EFTF1 and EFTF2. The sample included US adults who were older than 55 years by 2020 (community dwelling and noninstitutionalized) and their spouse or partner [35]. Telephone interviews were conducted to collect the data due to social contact restriction during the pandemic [32]. The data collection started on June 11, 2020, for EFTF1 and on September 24, 2020, for EFTF2. The current data were originally released in November 2020 and updated in February 2021 [32]. While data collection for both samples is still under way, the current data include 3266 respondents from the EFTE1 sample, with a response rate of 62% [32]. The HRS data set was approved for use without seeking institutional review board approval by the first author’s institution.

### Approaches

#### Dependent Variable

The outcome variable was depression, and it was measured by Composite International Diagnostic Interview-Short Form (CIDI-SF) [36,37]. In CIDI-SF, participants were asked 2 series of stem questions, with one containing questions about having 2 weeks of dysphoria (series A) and the other containing questions about having 2 weeks of anhedonia (series B) as follows: (A1) During the last 12 months, was there ever a time when you felt sad, blue, or depressed for 2 weeks or more in a row? (A2) Thinking of the 2-week period during the last 12 months when these feelings were worst, did the feelings of being sad, blue, or depressed usually last all day long, most of the day, about half the day, or less than half the day? (A3) Thinking of the 2-week period during the last 12 months when you felt sad, blue, or depressed, did you feel this way every day, almost every day, or less often than that? (B1) During the last 12 months, was there ever a time lasting 2 weeks or more when you lost interest in most things like hobbies, work, or activities that usually give you pleasure? (B2) Thinking of the 2-week period during the last 12 months when you had the most complete loss of interest in things, did the loss of interest usually last all day long, most of the day, about half the day, or less than half the day? (B3) Thinking of the 2-week period during the last 12 months when you lost interest in most things, did you feel this way every day, almost every day, or less often than that?

Participants are considered to meet the diagnostic requirement for major depression (MD) if they report 2 weeks of the aforementioned symptoms in ether series (A or B) lasting at least most of the day and at least almost every day. In this study, 291 participants were identified as meeting the requirement.

Seven additional questions (yes or no) on symptoms are asked to participants who meet the diagnostic requirements as follows: losing interest, feeling tired, change in weight, trouble with sleep, trouble concentrating, feeling down, and thoughts about death (0=no, 1=yes). In this study, an MD score was obtained by summing up the above 7 items (range 0-7). Participants who reported three or more symptoms (MD score ≥3) are classified as MD probable cases. The final outcome variable “probable MD” was generated by integrating the 2 series of stem questions and 7 additional symptom questions, where “no” represented that participants did not have probable MD (not meeting the diagnostic stem requirement or having an MD score <3) and “yes” represented that participants had probable MD. Depression was analyzed as a binary variable (0=no, 1=yes).

#### Independent Variables

Four types of delayed care were independent variables. Participants were asked “Since March 2020, was there any time when you needed medical or dental care, but delayed getting it, or did not get it at all?” For participants who reported delayed care, another question was asked about the type of care that was delayed, including surgery, seeing the doctor, filling a prescription, and dental care. Combining both questions, 4 dummy variables were generated for delayed surgery, delayed seeing a doctor, delayed dental care, and delayed other care (0=no, 1=yes). Participants who reported delayed care were also asked the reasons for not getting care (could not afford it; could not get an appointment; the clinic/hospital/doctor’s office cancelled, closed, or suggested rescheduling; decided it could wait; was afraid to go; and other reasons).

#### Covariates

##### Demographic Characteristics

Demographic characteristics were included and analyzed as binary variables as follows: gender (0=male, 1=female), education (0=high school graduate or below, 1=some college or above), race (0=White/Caucasian, 1=Black/African American, 2=other), and ethnicity (0=non-Hispanic, 1=Hispanic).

##### Health Status

Self-reported health status, physical chronic conditions, self-reported pain, and pain medication use were included. Self-reported health status was measured with a 5-point scale in the survey (1=poor, 2=fair, 3=good, 4=very good, and 5=excellent) and was analyzed as a continuous variable. For physical chronic conditions, participants were asked whether a doctor had ever told them that they have the following medical conditions: high blood pressure or hypertension, diabetes or high blood sugar, cancer or a malignant tumor (excluding minor skin cancer), chronic lung disease, heart problems (ie, heart attack, coronary heart disease, angina, or congestive heart failure), stroke, arthritis or rheumatism, and high blood cholesterol levels. Participants responded yes or no to each condition (0=no, 1=yes). The final variable for physical chronic conditions was obtained by adding up responses for 8 conditions, and it was analyzed as a continuous variable. Self-reported pain was analyzed as a continuous variable that ranged from 0 to 3 (0=not troubled by pain at all, 1=mild pain, 2=moderate pain, 3=severe pain). Self-reported health status (range 1-5), physical chronic condition (range 0-8), and self-reported pain (range 0-3) were analyzed as continuous variables. Regarding pain medication use, participants who responded that they had taken over-the-counter pain medications or opioids (Vicodin, oxycodone [OxyContin], codeine, and morphine) for the treatment of pain were considered as using pain medications. Pain medication use was analyzed as a binary variable (0=no, 1=yes).

##### Pandemic Stressors

Pandemic stressors included perceived less often in-person contact, perceived more loneliness, having anyone known die from COVID-19, financial difficulty, and COVID concern, as these stressors have been reported in recent studies as factors associated with depression during the pandemic [7,10,13,14,38]. Perceived less often in-person contact and perceived more loneliness were analyzed as binary variables (0=no, 1=yes). Participants were asked “Has anyone you know died from COVID-19?” (0=no, 1=yes). To measure financial difficulty, participants were asked “How difficult is it for (you/your family) to meet monthly payments on (your/your family’s) bills?” with 5-point responses (1=not difficult at all, 5=completely difficult). For COVID-19 concern, participants were asked “Overall, on a scale from 1 to 10, where 1 is the least concerned and 10 is the most concerned, how concerned are you about the coronavirus pandemic?” Financial difficulty (range 1-5) and COVID-19 concern (range 1-10) were analyzed as continuous variables.

### Statistical Analysis

Univariate analyses were conducted to describe demographic characteristics, health status, pandemic stressors, and delayed care, as well as depression among participants. Bivariate analyses were used to examine the age difference for all variables between middle-aged adults (46-64 years old) and older adults (≥65 years old), and the unadjusted relationship between depression and all variables. Lastly, binary logistic regression was applied to examine the factors associated with depression, and especially investigate the association of delayed care with depression. All statistical analyses were conducted using Stata/SE 15.1 (StataCorp).

## Results

### Description of Demographic Characteristics, Health Status, Pandemic Stressors, and Delayed Care

According to Table 1, slightly more than half of the participants were older than 65 years (n=1890, 58.2%), were female (n=1137, 56.8%), had an education level of some college or above (n=1134, 56.6%), and were White (n=1146, 57.5%). Moreover, 26.1% (n=520) of participants were African American. The majority of participants were non-Hispanic (n=1583, 79.2%). In terms of the health status, participants reported a moderate health status (mean 3.08, range 1-5). The average number of physical chronic conditions among participants was more than two (mean 2.42, range 0-8). Participants reported a low level of pain (mean 0.78, range 0-3), and 68.6% (n=2214) of participants reported using over-the-counter medication or opioids for pain relief. Regarding pandemic stressors, 26.1% (n=542) of participants reported that they felt lonelier during the pandemic. Moreover, 38.9% (n=813) of participants perceived that they had less often in-person contact with others outside of the household during the pandemic, and 19.8% (n=641) of participants had someone they knew die from COVID-19. Participants reported a low level of financial difficulty (mean 1.76, range 1-5) and a high level of COVID concern (mean 7.78, range 1-10). Only few participants (n=135, 4.2%) had experienced delayed surgery since March 2020. Furthermore, 17.4% (n=560) of participants experienced delay seeing a doctor, and 21.8% (n=704) of participants experienced delayed dental care. Only 6.8% (n=220) of participants experienced delayed other care besides surgery, seeing a doctor, and dental care. Table 1 shows that middle-aged and older adults had significant differences in gender (*P*=.02), race (*P*=.002), physical chronic conditions (*P*<.001), self-reported pain (*P*=.049), pain medication use (*P*=.043), having anyone they know die from COVID (*P*<.001), financial difficulty (*P*<.001), COVID concern (*P*=.02), and delayed care (surgery, *P*=.02; seeing a doctor, *P*<.001; dental care, *P*<.001; and other care, *P*<.001).

**Table 1:** Demographic characteristics, health status, pandemic stressors, and delayed care by age.

Characteristic				All (N=3246^a^)		Age 30-64 years (n=1356, 41.8%)		Age ≥65 years (n=1890, 58.2%)		*P* value^b^
**Demographics**										
	**Gender, n (%)**								.02	
		Male	866 (43.2)		540 (41.3)		326 (46.9)			
		Female	1137 (56.8)		768 (58.7)		369 (53.1)			
	**Education, n (%)**								.96	
		High school graduate or below	869 (43.4)		568 (43.4)		301 (43.3)			
		Some college or above	1134 (56.6)		740 (56.6)		394 (56.7)			
	**Race, n (%)**								.002	
		White/Caucasian	1146 (57.5)		719 (55.3)		427 (61.8)			
		Black/African American	520 (26.1)		344 (26.4)		176 (25.5)			
		Other	326 (16.4)		238 (18.3)		88 (12.7)			
	**Ethnicity, n (%)**								.84	
		Non-Hispanic	1583 (79.2)		1036 (79.3)		547 (78.9)			
		Hispanic	416 (20.8)		270 (20.7)		146 (21.1)			
**Health status**										
	Self-reported health status (range 1-5), mean (SD)		3.08 (1.01)		3.09 (1.04)		3.08 (1.01)		.64	
	Physical chronic conditions (range 0-8), mean (SD)		2.42 (1.53)		1.95 (1.46)		2.75 (1.49)		<.001	
	Self-reported pain (range 0-3), mean (SD)		0.78 (1.03)		0.82 (1.06)		0.75 (1.01)		.049	
	**Pain medication use, n (%)**								.043	
		No	1014 (31.4)		398 (29.3)		616 (32.8)			
		Yes	2214 (68.6)		953 (70.7)		1261 (67.2)			
**Pandemic stressors**										
	**Perceived more loneliness, n (%)**								.80	
		No	1537 (73.9)		558 (73.6)		979 (74.1)			
		Yes	542 (26.1)		200 (26.4)		342 (25.9)			
	**Perceived less in-person contact, n (%)**								.91	
		No	1276 (61.1)		463 (60.9)		813 (61.2)			
		Yes	813 (38.9)		297 (39.1)		516 (38.8)			
	**Anyone they know died from COVID, n (%)**								<.001	
		No	2593 (80.2)		1022 (75.5)		1571 (83.5)			
		Yes	641 (19.8)		331 (24.5)		310 (16.5)			
	Financial difficulty (range 1-5), mean (SD)		1.76 (0.94)		1.98 (1.01)		1.63 (0.87)		<.001	
	COVID concern (range 1-10), mean (SD)		7.78 (2.66)		7.65 (2.71)		7.87 (2.62)		.02	
**Delayed care**										
	**Delayed surgery, n (%)**								.02	
		No	3091 (95.8)		1281 (94.8)		1810 (96.5)			
		Yes	135 (4.2)		70 (5.2)		65 (3.5)			
	**Delayed seeing a doctor, n (%)**								<.001	
		No	2665 (82.6)		1053 (77.9)		1612 (86.1)			
		Yes	560 (17.4)		299 (22.1)		261 (13.9)			
	**Delayed dental care, n (%)**								<.001	
		No	2526 (78.2)		999 (73.8)		1527 (81.4)			
		Yes	704 (21.8)		354 (26.2)		350 (18.6)			
	**Delayed other care, n (%)**								<.001	
		No	3009 (93.2)		1236 (91.3)		1773 (94.6)			
		Yes	220 (6.8)		118 (8.7)		102 (5.4)			

^a^The total sample size of the study may not be the same as the total sample size of the survey due to missing values.

^b^We performed the *t* test for continuous variables and the χ^2^ test for categorical variables.

### Reasons for Delayed Care

As shown in Table 2, the clinic/hospital/doctor’s office cancelled, closed, or suggested rescheduling was the most common reason for delayed care (n=423, 43.8%). Moreover, around 10% of participants delayed care because they could not afford it (n=92, 9.5%), could not get an appointment (n=97, 10.0%), or were afraid to go (n=88, 9.1%). Slightly more than 13% of participants reported that they decided to wait (n=133, 13.8%), and the same percentage of participants delayed care for other reasons (n=133, 13.8%). The 2 age groups showed a significant difference in the reasons for delayed care (*P*=.002). More middle-aged adults reported that they delayed care because they could not afford it or they were afraid to go, while more older adults reported reasons, including the clinic/hospital/doctor’s office cancelled, closed, or suggested rescheduling, could not get an appointment, decided to wait, or other reasons.

**Table 2:** Reasons for delayed care.

Characteristic	All (N=968^a^), n (%)	Age 30-64 years, n (%)	Age ≥65 years, n (%)	*P* value
Could not afford it	92 (9.5)	62 (12.8)	30 (6.2)	.002
Could not get an appointment	97 (10.0)	57 (11.8)	40 (8.3)	.002
The clinic/hospital/doctor’s office cancelled, closed, or suggested rescheduling	423 (43.8)	200 (41.3)	223 (46.7)	.002
Decided it could wait	133 (13.8)	62 (12.8)	71 (14.7)	.002
Afraid to go	88 (9.1)	45 (9.3)	43 (8.9)	.002
Other reasons	133 (13.8)	58 (12.0)	75 (15.6)	.002

^a^The total sample size of the study may not be the same as the total sample size of the survey due to missing values.

### Depression Among US Middle-Aged and Older Adults During the Pandemic

**Figure 1:**
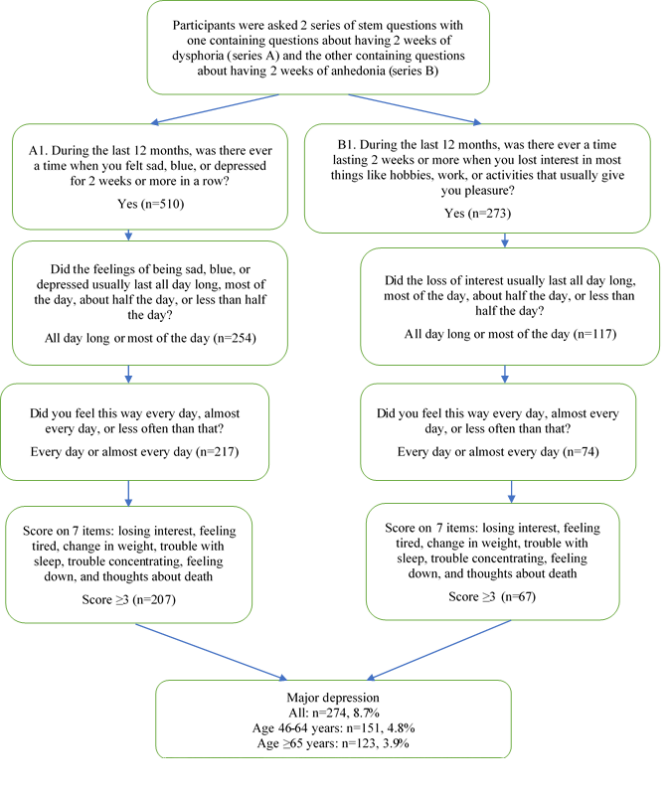
Depression among US middle-aged and older adults during the pandemic: Composite International Diagnostic Interview-Short Form (CIDI-SF).

As shown in Figure 1, for the first series of CIDI-SF stem questions (series A), 510 participants reported that they felt sad, blue, or depressed for 2 weeks or more in a row during the last 12 months. Of those participants, 254 reported that when these feelings were the worst, the feelings lasted for most of the day or all day long. Of the 254 participants, 217 reported that they had the feelings every day or almost every day. Among the 217 participants, 207 reported scores higher than 3 on the 7 symptom questions. For the second series of CIDI-SF stem questions (series B), 273 participants reported that they lost interest in most things like hobbies, work, or activities that usually give them pleasure for 2 weeks or more in a row during the last 12 months. Of those participants, 117 reported that when they completely lost interest in most things, the feelings lasted for most of the day or all day long. Of the 117 participants, 74 reported that they had the feelings every day or almost every day. Among the 74 participants, 67 reported scores higher than 3 on the 7 symptom questions. Totally, 274 (8.8%) participants were identified as having MD. Table 3 shows that participants with MD and participants without MD had significant differences in age (*P*<.001), gender (*P*<.001), self-reported health status (*P*<.001), physical chronic conditions (*P*<.001), self-reported pain (*P*<.001), pain medication use (*P*<.001), perceived less in-person contact with others (*P*=.001), financial difficulty (*P*<.001), COVID concern (*P*=.009), and delayed care (surgery, *P*<.001; seeing a doctor, *P*<.001; dental care, *P*<.001; and other care, *P*<.001). The unadjusted bivariate analysis indicated that participants with depression tended to be middle-aged and female, had worse self-reported health status, had more physical chronic conditions, perceived more loneliness, perceived less in-person contact with others, had financial difficulty, had a higher COVID concern score, had delayed care (surgery, seeing a doctor, dental care, and other care), and reported worse pain and pain medication use.

**Table 3:** Demographic characteristics, health status, pandemic stressors, and delayed care by depression.

Characteristics				All (N=3246^a^)		Participants without major depression (n=2840, 91.2%)		Participants with major depression (n=274, 8.8%)		*P* value^b^
**Demographics**										
	**Age, n (%)**									<.001
		30-64 years	1356 (41.8)		1179 (41.5)		151 (55.1)			
		≥65 years	1890 (58.2)		1661 (58.5)		123 (44.9)			
	**Gender, n (%)**									<.001
		Male	866 (43.2)		774 (43.9)		62 (31.0)			
		Female	1137 (56.8)		990 (56.1)		138 (69.0)			
	**Education, n (%)**									.97
		High school graduate or below	869 (43.4)		759 (43.0)		88 (44.0)			
		Some college or above	1134 (56.6)		1005 (57.0)		112 (56.0)			
	**Race, n (%)**									.98
		White/Caucasian	1146 (57.5)		1011 (57.6)		114 (57.3)			
		Black/African American	520 (26.1)		456 (26.0)		53 (26.6)			
		Other	326 (16.4)		287 (16.4)		32 (16.1)			
	**Ethnicity, n (%)**									.38
		Non-Hispanic	1583 (79.2)		1386 (78.7)		162 (81.4)			
		Hispanic	416 (20.8)		375 (21.3)		37 (18.6)			
**Health status**										
	Self-reported health status (range 1-5), mean (SD)			3.08 (1.01)		3.17 (1.98)		2.48 (1.05)		<.001
	Physical chronic conditions (range 0-8), mean (SD)			2.42 (1.53)		2.35 (1.50)		3.04 (1.64)		<.001
	Self-reported pain (range 0-3), mean (SD)			0.78 (1.03)		0.70 (0.98)		1.49 (1.18)		<.001
	**Pain medication use, n (%)**									<.001
		No	1014 (31.4)		913 (32.3)		57 (20.9)			
		Yes	2214 (68.6)		1913 (67.7)		216 (79.1)			
**Pandemic stressors**										
	**Perceived more loneliness, n (%)**									<.001
		No	1537 (73.9)		1424 (75.5)		79 (52.7)			
		Yes	542 (26.1)		462 (24.5)		71 (47.3)			
	**Perceived less in-person contact, n (%)**									.001
		No	1276 (61.1)		1173 (61.8)		72 (48.3)			
		Yes	813 (38.9)		724 (38.2)		77 (51.7)			
	**Anyone they know died from COVID, n (%)**									.85
		No	2593 (80.2)		2262 (80.0)		217 (79.5)			
		Yes	641 (19.8)		567 (20.0)		56 (20.5)			
	Financial difficulty (range 1-5), mean (SD)			1.76 (0.93)		1.72 (0.90)		2.21 (1.21)		<.001
	COVID concern (range 1-10), mean (SD)			7.86 (2.59)		7.82 (2.59)		8.25 (2.50)		.009
**Delayed care**										
	**Delayed surgery, n (%)**									<.001
		No	3091 (95.8)		2723 (96.4)		241 (89.3)			
		Yes	135 (4.2)		103 (3.6)		29 (10.7)			
	**Delayed seeing a doctor, n (%)**									<.001
		No	2665 (82.6)		2369 (83.9)		174 (64.2)			
		Yes	560 (17.4)		455 (16.1)		97 (35.8)			
	**Delayed dental care, n (%)**									<.001
		No	2526 (78.2)		2233 (79.0)		172 (63.5)			
		Yes	704 (21.8)		595 (21.0)		99 (36.5)			
	**Delayed other care, n (%)**									<.001
		No	3009 (93.2)		2657 (94.0)		223 (82.3)			
		Yes	220 (6.8)		170 (6.0)		48 (17.7)			

^a^The total sample size of the study may not be the same as the total sample size of the survey due to missing values.

^b^We performed the *t* test for continuous variables and the χ^2^ test for categorical variables.

### Factors Associated With Depression Among Middle-Aged and Older Adults

Table 4 shows that after controlling demographics, health status, and pandemic stressors, delayed dental care was positively associated with depression among both middle-aged adults (odds ratio [OR] 2.05, 95% CI 1.04-4.03; *P*=.04) and older adults (OR 3.08, 95% CI 1.07-8.87; *P*=.04). The results indicated that participants who reported delayed dental care had a higher log OR of having depression compared to those who did not. Moreover, delayed surgery was positively associated with depression among older adults (OR 3.69, 95% CI 1.06-12.90; *P*=.04).

Other factors associated with depression are also reported in Table 4. Self-reported pain was positively related to depression among both middle-aged adults (OR 1.74, 95% CI 1.28-2.37; *P<*.001) and older adults (OR 1.98, 95% CI 1.22-3.21; *P*=.005). Middle-aged adults who reported higher education levels (some college or above) (OR 2.98, 95% CI 1.06-4.11; *P*=.03) or worse self-reported health (OR 0.68, 95% CI 0.47-0.98; *P*=.04) had a higher likelihood of having depression. While perceived more loneliness was positively associated with depression among older adults (OR 3.58, 95% CI 1.24-10.29; *P*=.02), financial difficulty was positively associated with depression among middle-aged adults (OR 1.78, 95% CI 1.33-2.37; *P<*.001).

**Table 4:** Binary logistic regression for the association of delayed care with depression on controlling covariates between the age groups.

Characteristic			Depression								
			Age 46-64 years^a^				Age ≥65 years^b^				
			OR (95% CI)	*P* value		OR (95% CI)			*P* value		
**Demographics**											
	Gender: female (reference: male)	1.43 (0.74-2.76)			.28			2.27 (0.87-5.93)		.10	
	Education: some college or above (reference: high school graduate or below)	2.98 (1.06-4.11)			.03			0.69 (0.28-1.71)		.42	
	Race: Black/African American (reference: White/Caucasian)	1.02 (0.48-2.19)			.96			0.81 (0.25-2.57)		.72	
	Race: other (reference: White/Caucasian)	1.20 (0.48-3.01)			.69			0.86 (0.15-5.05)		.87	
	Ethnicity: Hispanic (reference: non-Hispanic)	0.73 (0.30-1.78)			.49			0.92 (0.24-3.58)		.90	
**Health status**											
	Self-reported health status	0.68 (0.47-0.98)			.04			0.82 (0.49-1.40)		.47	
	Physical chronic condition	1.10 (0.88-1.37)			.40			0.89 (0.64-1.23)		.49	
	Self-reported pain	1.74 (1.28-2.37)			<.001			1.98 (1.22-3.21)		.005	
	Pain medication use: yes (reference: no)	1.34 (0.54-3.33)			.53			0.41 (0.16-1.06)		.07	
**Pandemic stressors**											
	Perceived more loneliness: yes (reference: no)	1.00 (0.48-2.10)			.99			3.58 (1.24-10.29)		.02	
	Perceived less in-person contact: yes (reference: no)	1.15 (0.55-2.37)			.71			1.20 (0.39-3.67)		.75	
	Anyone they know died from COVID: yes (reference: no)	0.55 (0.24-1.25)			.16			1.13 (0.40-3.19)		.82	
	Financial difficulty	1.78 (1.33-2.37)			<.001			0.92 (0.64-1.41)		.70	
	COVID concern	0.94 (0.84-1.06)			.31			0.97 (0.80-1.18)		.77	
**Delayed care**											
	Delayed surgery: yes (reference: no)	0.40 (0.12-1.31)			.13			3.69 (1.06-12.90)		.04	
	Delayed seeing a doctor: yes (reference: no)	1.24 (0.60-2.57)			.57			1.78 (0.62-5.09)		.29	
	Delayed dental care: yes (reference: no)	2.05 (1.04-4.03)			.04			3.08 (1.07-8.87)		.04	
	Delayed other care: yes (reference: no)	2.02 (0.88-4.64)			.10			0.38 (0.07-2.12)		.27	

^a^The number of observations was 697, log likelihood was −158.90, LR chi-square (19) was 95.88 (*P*<.001), and pseudo R-square was 0.2318.

^b^The number of observations was 437, log likelihood was −89.89, LR chi-square (19) was 44.01 (*P*<.001), and pseudo R-square was 0.1966.

## Discussion

### Principal Results and Comparison to Prior Work

This study used national representative survey data from the HRS and aimed to (1) assess the prevalence of depression and delayed care among US middle-aged and older adults during the COVID-19 pandemic and (2) examine factors associated with depression among those 2 age groups during the pandemic, and particularly investigate the association of delayed care with depression. This study is the first to examine and compare the association of delayed care with depression among middle-aged and older adults in the United States during the COVID-19 pandemic.

#### Depression and Delayed Care

For the first aim, this study found that 274 (8.7%) participants reported symptoms for depression. Using different measures for depression, previous studies have reported high depression levels among US adults during the pandemic [8-11]. Two studies compared 2 nationally representative surveys of US adults, and both concluded that depression levels measured by the PHQ (PHQ-2 or PHQ-9) were higher during the pandemic than in 2017-2018 [6,7].

This study also found that about 17% of participants delayed seeing a doctor and about 20% of participants had delayed dental care, while few participants had delayed surgery and other medical care. Although statistics of delayed care among the same sample before the pandemic are not available, a recent study reported that emergency department visits significantly declined after the declaration of the COVID-19 national emergency [24]. A web-based survey in June 2020 reported that 40.9% of US adults delayed medical care since the pandemic, with 12% delaying emergency care and 31.5% delaying routine care [25]. The most common reason for delayed care in this study was that the clinic/hospital/doctor’s office cancelled, closed, or suggested rescheduling. A previous study also suggested that the increased delayed care might be associated with the social distancing policy or participants’ concerns about COVID-19 [23].

#### Association of Delayed Care With Depression

With regard to the second aim, delayed dental care was positively associated with depression in both middle-aged and older adults. This relationship was not documented in previous studies. Considering the positive relationship between untreated or undertreated pain and depression, delayed dental care might be linked to depression through pain caused by dental issues. A previous study also reported that dental pain was positively associated with depression [39]. Another study also suggested that having an oral health condition was positively linked to depression among adults [40]. Moreover, delayed surgery was positively related to depression among older adults but not middle-aged adults. Only a single previous study examined the association between delayed care and depression during the pandemic [26]. Recent studies indicated that untreated or undertreated pain from delayed medical care might be prevalent during the pandemic [27,28]. Meanwhile, a previous study reported that patients experienced higher levels of depression during a 3-month wait time for pain treatment than when their pain was treated [29]. Given the strong link between depression and pain, Kaiser pointed out that delayed surgery that would reduce pain and suffering during the pandemic might make patients’ conditions worse and increase depression [41]. Compared to middle-aged adults, older adults reported more chronic physical conditions, which might explain the higher demand on surgery among older adults and the insignificant relationship between delayed surgery and depression among middle-aged adults.

#### Other Factors Associated With Depression

This study also found that self-reported pain was positively linked to depression among both age groups. The positive relationship between pain and depression was also frequently reported in previous literature [42-45]. Patients with pain might experience undertreatment of pain or untreated pain during the pandemic [27,28], which was found to be associated with significantly higher depression levels compared to when the pain was treated [29]. In addition, education, self-reported health status, and financial difficulty were significantly associated with depression only among middle-aged adults, while perceived more loneliness was significantly related to depression only among older adults. Middle-aged adults with higher educational levels (some college or above) were more likely to have depression in this study, which is not consistent with previous studies that reported a negative relationship between educational levels and depression [46,47]. This inconsistent finding implies that middle-aged adults with higher education might be impacted by the pandemic disproportionally, though the path is not known yet. Middle-aged adults reporting worse health were more likely to have depression, which is consistent with previous studies [48-50]. Moreover, middle-aged adults with higher levels of financial difficulty had a higher risk of depression, which is in line with previous studies that were conducted among general US adults during the pandemic [9,51,52]. While younger adults or middle-aged adults might lose their jobs during the pandemic and face financial difficulty because of unemployment, older adults aged 65 years or over are less likely to be in the labor force and therefore less likely to be impacted financially by the pandemic [53]. The study also found that older adults who perceived more loneliness during the pandemic were more likely to get depressed. This is consistent with previous studies, which indicated that perceived loneliness was positively associated with depression among older adults during the pandemic [54-56]. Although such a relationship was also documented by studies conducted among general adults [57] and younger adults aged 22 to 29 years [58], this study did not find a significant relationship between perceived more loneliness and depression among middle-aged adults.

### Limitations

There were several limitations in this study. First, the cross-sectional design of this study was not able to examine the causal effect of delayed care on depression during the pandemic. Second, delayed care was simply measured by 4 yes-or-no questions, and the details of delayed care, such as the urgency of the care needed and how long the care was delayed, were not known. More comprehensive measurements for delayed care are needed in a future study to examine the path through which delayed care is associated with depression. Third, the data collection for this study happened during fall and summer 2020, while the peak of COVID cases/deaths occurred during winter 2020-2021 (November 2020 to January 2021) [59]. Therefore, the delayed care during the pandemic might have been underestimated.

### Implications for Practice and Research

While previous studies focused on examining depression among younger adults or general adults during the pandemic [7,9-14,38,52], this study found that depression was also prevalent among middle-aged and older adults during the pandemic. Delayed surgery was positively associated with depression among older adults, and delayed dental care was significantly associated with depression among both middle-aged and older adults. Despite the limitations, this study has several implications for future practice and research. First, literature regarding the association of delayed care with depression during the pandemic is limited, and future research that uses more comprehensive measurements for delayed care is needed to decode the path through which delayed care is associated with depression. Second, this study highlighted the collateral damage of the COVID-19 pandemic by identifying the association of delayed surgery and dental care with depression during the pandemic, which provides evidence for the assessment of the indirect effect of the COVID-19 pandemic on non-COVID–related health [23]. Third, although surgery and dental care cannot be delivered by telehealth, telehealth services can still be provided to address patients’ concerns on delayed surgery and dental care. Moreover, the implementation of telemental health services is needed to address mental health symptoms among US middle-aged and older adults during the pandemic [26].

## Abbreviations

CIDI-SF: Composite International Diagnostic Interview-Short Form
EFTF: enhanced face-to face interviewing
HRS: Health and Retirement Study
MD: major depression
PHQ: Patient Health Questionnaire
